# The Renin-Angiotensin System: The Challenge behind Autoimmune Dermatological Diseases

**DOI:** 10.3390/diagnostics13223398

**Published:** 2023-11-07

**Authors:** Minela Aida Maranduca, Mihai Andrei Cosovanu, Andreea Clim, Alin Constantin Pinzariu, Nina Filip, Ilie Cristian Drochioi, Vlad Ionut Vlasceanu, Daniel Vasile Timofte, Roxana Nemteanu, Alina Plesa, Mihaela Pertea, Ionela Lacramioara Serban

**Affiliations:** 1 Discipline of Physiology, Department of Morpho-Functional Sciences II, “Grigore T. Popa” University of Medicine and Pharmacy, 700115 Iasi, Romania; 2 Internal Medicine Clinic, “St. Spiridon” County Clinical Emergency Hospital, 700115 Iasi, Romania; 3 Discipline of Biochemistry, Department of Morpho-Functional Sciences II, “Grigore T. Popa” University of Medicine and Pharmacy, 700115 Iasi, Romania; 4 Department of Oral and Maxillofacial Surgery and Reconstructive, Faculty of Dental Medicine, “Grigore T. Popa” University of Medicine and Pharmacy, 700020 Iasi, Romania; 5 Discipline of Surgical Semiology, Department of Surgery I, “Grigore T. Popa” University of Medicine and Pharmacy, 700115 Iasi, Romania; 6 Medical I Department, “Grigore T. Popa” University of Medicine and Pharmacy, 700115 Iasi, Romania; 7 Institute of Gastroenterology and Hepatology, “St. Spiridon” University Hospital, 700111 Iasi, Romania; 8 Department of Plastic Surgery and Reconstructive Microsurgery, “Sf. Spiridon” Emergency County Hospital, 700111 Iasi, Romania

**Keywords:** RAS, autoimmune diseases, dermatology, psoriasis, systemic sclerosis, vitiligo, lupus erythematosus

## Abstract

Autoimmune dermatological diseases (AIDD) encompass a diverse group of disorders characterized by aberrant immune responses targeting the skin and its associated structures. In recent years, emerging evidence suggests a potential involvement of the renin–angiotensin system (RAS) in the pathogenesis and progression of these conditions. RAS is a multicomponent cascade, primarily known for its role in regulating blood pressure and fluid balance. All of the RAS components play an important role in controlling inflammation and other immune responses. Angiotensin II, the main effector, acts on two essential receptors: Angiotensin Receptor 1 and 2 (AT1R and AT2R). A disturbance in the axis can lead to many pathological processes, including autoimmune (AI) diseases. AT1R activation triggers diverse signaling cascades involved in inflammation, fibrosis and tissue remodeling. Experimental studies have demonstrated the presence of AT1R in various cutaneous cells and immune cells, further emphasizing its potential contribution to the AI processes in the skin. Furthermore, recent investigations have highlighted the role of other RAS components, beyond angiotensin-converting enzyme (ACE) and Ang II, that may contribute to the pathophysiology of AIDD. Alternative pathways involving ACE2, Ang receptors and Ang-(1-7) have been implicated in regulating immune responses and tissue homeostasis within the skin microenvironment. Understanding the intricate involvement of the RAS in AIDD may provide novel therapeutic opportunities. Targeting specific components of the RAS, such as angiotensin receptor blockers (ARBs), ACE inhibitors (ACEIs) or alternative RAS pathway modulators, could potentially ameliorate inflammatory responses, reduce tissue damage and lessen disease manifestations. Further research is warranted to outline the exact mechanisms underlying RAS-mediated immune dysregulation in AIDD. This abstract aims to provide a concise overview of the intricate interplay between the RAS and AIDD. Therefore, we elaborate a systematic review of the potential challenge of RAS in the AIDD, including psoriasis, systemic sclerosis, vitiligo, lupus erythematosus and many more.

## 1. Introduction

AIDD are a group of conditions that arise when the immune system erroneously attacks healthy skin cells. Some common AIDD include psoriasis, vitiligo, lupus erythematosus, scleroderma, alopecia areata, bullous pemphigoid, lichen planus and pemphigus. Traditionally, the afore-mentioned conditions have been attributed to various factors, including RAS.

RAS is a complex system with diverse functions beyond autoimmunity, and more research is needed to fully understand its role in AI diseases. Chronic inflammation is a hallmark of many AI diseases, and Ang II’s pro-inflammatory effects may contribute to the development or progression of these conditions. Moreover, Ang II can stimulate the activation of immune cells, which are crucial in the immune responses [1]. This activation can induce the generation of pro-inflammatory molecules that contribute to the AI process. The aim of this study is to decipher the interaction between the RAS and AIDD, which could provide opportunities for targeted therapeutic strategies [1,2].

## 2. Materials and Methods

The literature search was conducted using electronic databases (PubMed, Scopus, SpringerLink, Wiley, Elsevier, ResearchGate and Google Scholar), focusing on studies published within the last 10 years. Our search queried “RAS [AND] autoimmune diseases [OR] dermatology [OR] psoriasis [OR] systemic sclerosis [OR] vitiligo [AND] lupus erythematosus“ and was limited only to prospective and retrospective studies and metanalyses, omitting abstracts, documents, and reviews. Our search resulted in 140 total references. Findings and insights from the selected studies were synthesized to provide a comprehensive overview of the role of RAS in AIDD.

## 3. The Renin–Angiotensin–Aldosterone System

**Figure 1:**
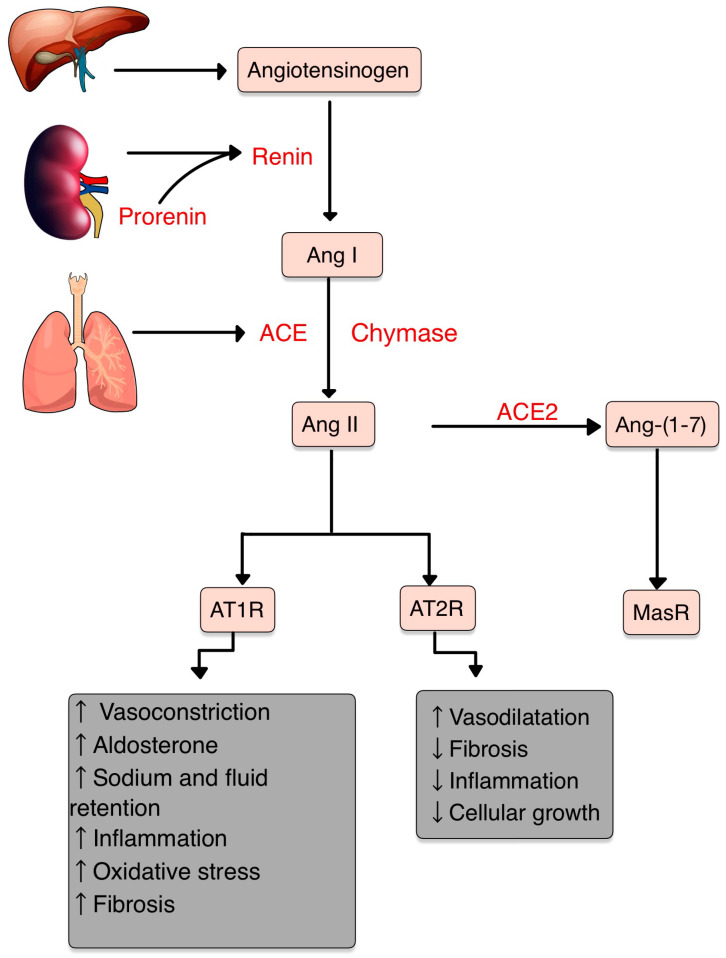
RAS and its components. Ang II, the pivotal element, acts mainly on AT1R and has opposite effects on AT2R. AT1R promotes vasoconstriction, inflammation, fibrosis, oxidative stress (OS) and increases the aldosterone levels. Contrarily, AT2R promotes vasodilatation and decreases inflammation and cellular growth. Ang II can be transformed by ACE2 into Ang-(1-7), which acts on MasR, with anti-inflammatory effects (adapted after [14]).

The central role of RAS is to maintain blood pressure and body fluid homeostasis [1]. The kidney is the main source of prorenin, the precursor of renin. Low arterial pressure, low sodium chloride and activation of beta-1 adrenoceptor lead to the release of renin from the juxtaglomerular cells [2]. Renin is a proteinase that hydrolyzes angiotensinogen (also called renin substrate), a plasma alfa-2-globulin synthesized by the liver and released into the blood flow. The Ang I resulting is a mild vasoconstrictor agent with no significant changes in blood pressure homeostasis. Thus, Ang I is further transformed into Ang II by ACE, present in lung capillaries, kidney and endothelial cells (ECs) (Figure 1) [3,4,5].

Chymase is a serine protease with a significant role in the conversion of Ang I into Ang II through a non-ACE pathway. This enzyme is found in mast cells, vascular ECs and cardiac fibroblasts, thereby serving as a primary contributor to the production of Ang II within the tissues [6,7].

Ang II, a very potent vasoconstrictor, exerts its effects through the activation of two G-protein-coupled receptors (GPCRs): AT1R and AT2R. AT2Rs appear especially in the fetal period, and their number decreases shortly after birth. Contrarily, AT1Rs appear mainly in the adult organism [4,8]. Natural antibodies (Abs) against GPCRs, involved in physiological homeostasis, including immune responses, have been identified in healthy individuals. However, when the levels or functions of these Abs become dysregulated, pathological mechanisms that contribute to the development of AI diseases, including systemic sclerosis, can result [9].

The Ang II-AT1R pathway is essential for survival [10]. In vascular smooth muscle cells (VSMCs), Ang II binds to AT1R, activating phospholipase C and raising intracellular [Ca^+2^], leading to vasoconstriction [3,4]. Activating AT2R, Ang II lowers blood pressure by vasodilatation and nitric oxide (NO) release [11]. Also, AT2R is involved in wound healing and tissue remodeling [12].

Aldosterone, the final element in the RAS, stimulates Na+ reabsorption [3,4]. Significantly, Aldosterone and Ang II are implicated in the production of extracellular matrix (ECM) proteins, including fibronectin, collagen I and plasminogen activator inhibitor proteins. These actions suggest an important role of RAS in tissue fibrosis [13].

## 4. RAS and Inflammation

The interaction with AT1R promotes the classic effects of inflammation, vasoconstriction, OS and increased proliferation factors [15,16]. On the other hand, AT2R, ACE2, Ang1-7, Ang1-9 and Mas Receptor (MasR) have anti-inflammatory properties [17,18].

The binding Ang II-AT1R enhances vascular permeability and synthesis of vascular endothelial growth factor (VEGF), stimulates adhesion molecule expression by neutrophils (PMNs) and ECs, including selectins (P- and L- selectin), VCAM-1, and ICAM-1 [10,16]. Likewise, the activation of AT1R induces inflammatory responses, such as the migration of leukocytes and the release of pro-inflammatory cytokines (e.g., IL-1β, IL6, TNF, CXCL-1) [17]. Ang II contributes to endothelial dysfunction by activating COX-2, resulting in the production of ROS and prostaglandins [10]. Peroxisome proliferator-activated receptors (PPARs) expression has been found to be suppressed by Ang II. This suppression leads to a decrease in the PPARs’ capabilities to mitigate inflammation [19].

In the presence of inflammatory cytokines or a tissue injury, the mast cells degranulate to the ECM and the chymase is activated. Ang I is converted to Ang II by the active chymase as well as ACE [20]. Chymase also activates MMP-9 and TGF-β by converting their inactive precursors into active forms, which are associated with OS, inflammation and fibrosis [6,21].

**Figure 2:**
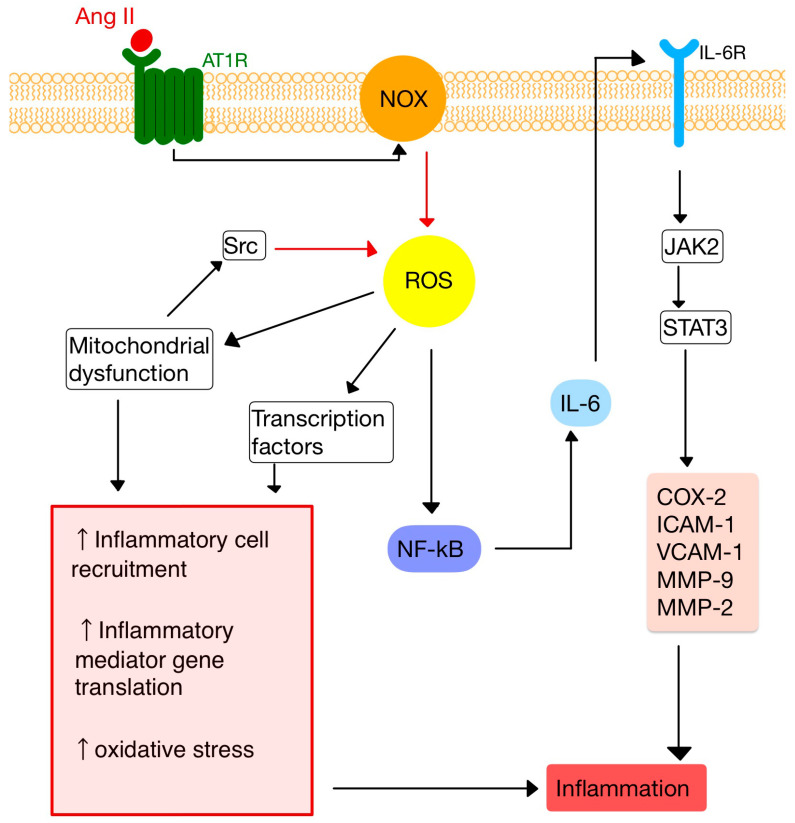
Ang II-mediated ROS activation. NF-kB, nuclear factor kappa B; JAK2, janus kinase 2; STAT3, signal transducer and activator of transcription 3; COX-2, cyclooxygenase-2; IL-6, interleukin-6 and its receptor, IL-6R; VCAM-1/ICAM-1, vascular cell/intercellular adhesion molecule 1; MMP-9, -2, matrix metalloproteinases -9, -2. (Adapted after [10,28]).

Ang II, by stimulating NADPH oxidases (NOX), induces reactive oxygen species (ROS) production, an important messenger involved in intracellular signaling (Figure 2) [10,22]. ROS are small molecules derived from oxygen metabolism, including hydrogen peroxide, superoxide, singlet oxygen and hydroxyl radical. Low ROS levels can adjust biological activities, such as cellular growth, differentiation, proliferation, signaling and senescence. However, when the ROS production is increased (OS), Ang II signaling is disturbed, causing endothelial dysfunction, vascular remodeling and inflammation [23,24,25,26].

Studies have shown that losartan (an ARB) suppresses Ang II-induced NF-κB activation, lowers the accumulation of VCAM-1, interrupts the functioning of toll-like receptors (TLR) 2 and 4, and also releases inflammatory mediators, such as IL-6 or C-reactive protein [27].

ROS have been implicated in the initiation, development and progression of psoriasis. Studies have indicated that in psoriasis there is a low activity of erythrocyte superoxide dismutase and catalase, while the levels of malondialdehyde, a NO end product, are elevated. These findings suggest a decrease in antioxidant capacity in psoriasis [29].

Ang II exerts multiple effects, including the stimulation of ROS production and cytokine release, which contribute to proinflammatory effects. The enzyme ACE inactivates bradykinin, which typically promotes vasodilation by generating NO, vascular permeability and proinflammatory cytokines synthesis, including IL-6 and IL-8. These cytokines, along with NO acting as a cytotoxic agent, have been implicated in the pathogenesis of vitiligo [30].

## 5. RAS and the Immune System

Inflammation is a vital mechanism for health. Through AT1R, the stimulation of ROS augments the inflammatory actions of the immune system [10,31].

**Figure 3:**
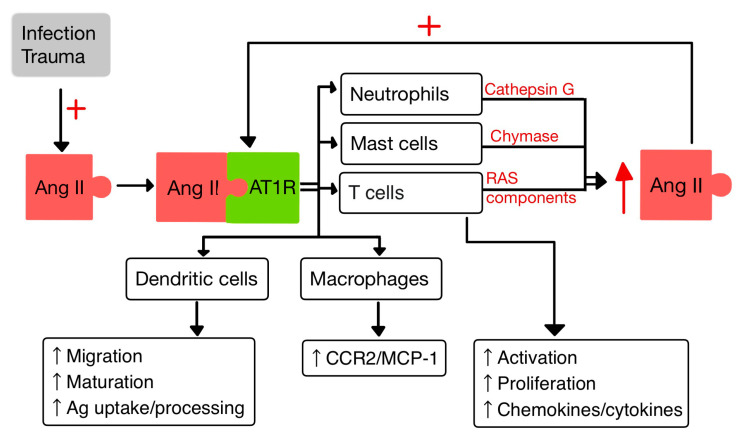
Interactions between Ang II and IIS (adapted after [10,14]).

The inflammatory response involves many cell types interacting with RAS: dendritic cells, T-cells, PMNs, mast cells and macrophages (Figure 3).

### 5.1. T Cells

T-cells hold an intrinsic RAS that regulates their migration and proliferation. In the context of inflammation, Ang II acts through the AT1R to induce rearrangements in the cytoskeleton of T cells. This activation leads to the release of chemokines and cytokines that enhance the recruitment of T cells to inflammatory sites [14]. The endogenously produced Ang II in T cells contributes to their activation, increases the production of TNF-α and upregulates the expression of C-C chemokine receptor 5 (CCR5) [10]. CCR5 plays an important role in recruiting and activating inflammatory cells [32]. In addition, TNF-α triggers various events, including the production of pro-inflammatory cytokines (IL-1, -6 and -8), adhesion molecules, generation of NO and release of pro-coagulatory substances. TNF-α acts on two receptors: TNFR1 and TNFR2 [33]. TNFR1 is involved in promoting pro-inflammatory and cytotoxic responses, while TNFR2 is primarily associated with proliferation, tissue regeneration and cell survival [34]. Furthermore, TNF-α can reduce the viability of antigen-presenting cells (APCs) [10].

Upon activation, native CD4+ T cells have the capacity to differentiate into two major subsets of T helper cells known as Th1 and Th2 [35]. Th1 cells contribute to cellular immunity, promote the killing efficiency of macrophages and stimulate the proliferation of CD8+ T cells. On the contrary, Th2 cells play a role in humoral immunity by stimulating the proliferation of B-cells and facilitating Abs class switching in B-cells [10,36]. It has been proved that the RAS may be involved in promoting Th1-mediated AI diseases [37]. Ang II has been found responsible for disrupting the Th1/Th2 balance by promoting the production of Th1 cytokine IFN-γ, thereby exerting pro-inflammatory effects, while reducing the levels of the Th2 cytokine IL-4 [38].

Tregs are tissue resident memory cells (TRM), which constitute approximately 20–40% of the CD4 T-cells in both human and mice skin. Their primary function is to uphold the immune homeostasis of the skin, facilitate wound healing and participate in tissue repair. These cells play an important role in chronic inflammatory conditions affecting the skin, such as psoriasis and vitiligo. Research conducted on mice and humans indicates that, in psoriasis, the imbalanced Th17/Tregs ratio implicated in the disease is influenced by the disfunction of Tregs in conjunction with the IL-23/IL-17 axis of inflammation [39].

### 5.2. Dendritic Cells

Dendritic cells (DCs) are specialized APCs that have a critical role in regulating the innate and also the adaptive immune responses [40,41].

In a study by Meng et al. [40], it was observed that Ang II exerts contrasting effects on DCs. On one hand, Ang II inhibits the phagocytic activity and proliferation of DCs. However, on the other hand, it promotes the maturation and the migration of DCs and also the expression of pro-inflammatory cytokines. Additionally, Ang II stimulates the T cell proliferation mediated by DCs [40]. Moreover, aldosterone enhances the capacity of DCs to activate CD8+ T cells response and to increase Th 17 polarization of CD4+ T cells. Also, Ald induces in DCs the secretion of IL-6 and TGF-β [41].

### 5.3. Macrophages

Macrophages and their precursors, known as monocytes, are white blood cells specialized in clearing away cellular debris and pathogens through phagocytosis. Additionally, they possess the ability to trigger and activate other immune cells to respond to invading pathogens [14].

Aldosterone aims at monocytes/macrophages and promotes the activation/migration of these cells in the ECs by increasing the expression of VCAM-1 and ICAM-1 [41].

It was proven that the activity of AT1R in M1 macrophages promotes polarization, which accelerates the inflammation with progression of tissue damage [42]. Likewise, Ang II upregulates the expression of monocyte chemoattractant protein-1 (MCP-1) and one of its receptors, CCR2 [14,43].

### 5.4. Neutrophils

PMNs, as the first responding cells to invading pathogens, play a crucial role in providing early immune protection. PMN bactericidal activity is increased by ACE present within them, regardless of the involvement of the Ang II/AT1R pathway. By interacting with AT1R, Ang II releases IL-8, which stimulates PMN recruitment and infiltration. Also, when stimulated by Ang II, PMNs produce oxidative bursts [10,44].

Cathepsin G (CatG), found in macrophages and PMNs, is a lysosomal protease that is upregulated in response to signals linked to infection and inflammation. CatG can elevate the local generation of Ang II by converting both angiotensinogen and Ang I to Ang II [10,45,46].

## 6. RAS in the Skin

New research findings have unveiled the presence of a local RAS within the skin, where ACE has a role in the regulation of inflammation and autoimmunity [47]. The components of the RAS are situated in cutaneous and subcutaneous layers. They are crucial in various skin-related conditions, such as inflammation, fibrosis, scar formation and certain types of skin cancers [48].

Components of RAS are expressed in the human skin. Initial studies highlighting the presence of a local RAS in the skin revealed that skin cells, particularly keratinocytes, possess the ability to produce Ang II (as well as potentially other angiotensins) independently of the systemic circulation’s supply of RAS components. Keratinocytes are abundant in AT1R throughout all epidermal layers. AT1 receptors are also expressed in the hair follicles and sweat glands. In dermis, fibroblasts express AT1R, AT2R, MAS, angiotensinogen, renin, ACE, Ang II, and mast cells express chymase. In hypodermis, the subcutaneous fat expresses the same components of RAS as in dermis plus ACE2 [49,50,51].

RAS activity is involved in cell proliferation, differentiation, tissue remodeling and skin photoaging [52,53]. Stimulation of AT1R triggers cellular processes, including cell proliferation, migration, collagen synthesis and angiogenesis. Contrarily, the AT2R inhibit these actions by blocking the synthesis of certain pro-inflammatory molecules, like TGF-β, TNF-α and IL-6. Therefore, the interplay between AT1R and AT2R within RAS provides a fragile balance in regulating these cellular functions and inflammatory responses in the skin [54].

**Figure 4:**
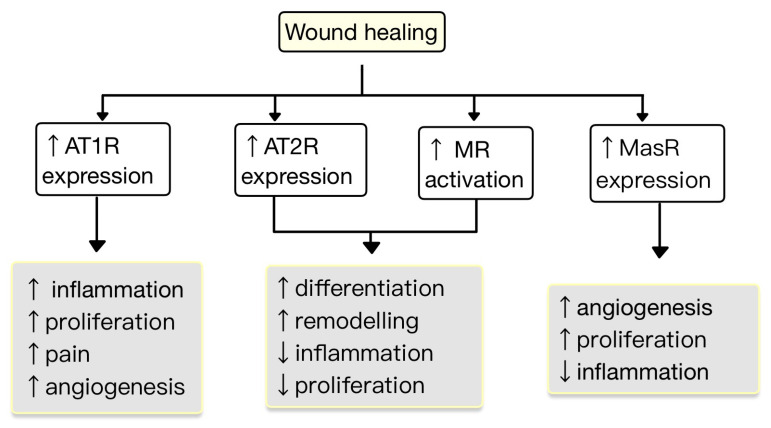
The role of RAS in the wound healing process. AT1R promotes inflammation, proliferation, angiogenesis and pain. AT2R and MR promote remodeling and differentiation and decrease inflammation and proliferation. MasR increases proliferation and angiogenesis and decreases inflammation (adapted after [49]).

The expression of RAS components is upregulated in human wounded skin. Ang receptors have been implicated in wound healing and scar formation of the skin (Figure 4) [49]. Impaired wound healing has been associated with disruptions in the function of AT1R. In both aging and diabetes, RAS dysregulation occurs, characterized by high AT1R expression and low AT2R expression. This modification in the AT1R/AT2R ratio is linked with a reduction in epidermal thickness, collagen degeneration, dermal layer fractures and subcutaneous fat atrophy [55]. Research studies revealed that valsartan (an ARB) exhibits the highest level of skin penetration among other ARBs. Topical application of 1% valsartan gel has shown significant enhancement in wound healing. Researchers found that the rate of wound healing is superior while using topical valsartan compared to losartan. The beneficial effects of valsartan gel were mediated through the activation of AT2R, as the healing effect was absent in mice lacking AT2R. Conversely, the application of a 5% captopril gel resulted in a notable delay in the process of wound healing [48].

Hypertrophic and keloids scars are characterized by an aberrant wound healing process that results in excessive ECM production. There is evidence that both Ang II and AT1R concentrations are elevated in keloid and hypertrophic scars compared to normal skin. AT1R promotes scar formation. In hypertrophic and keloid scars, the AT1R activation leads to increased ECM production, transition of fibroblasts into myofibroblasts and contraction of granulation tissue. This process involves the activation of TGF-β signaling pathways. Elevated levels of Ang II, acting through AT1R, contribute to skin scar formation by upregulating the expression of inflammatory molecules (e.g., IL-6, VEGF, TGF-β1) [48,51,54].

ACEIs reduce scar formation, inhibit fibroblast proliferation, and suppress the expression of TGF-β1 and collagen. TGF-β1 has cytoprotective effects in mitigating tissue injury through promoting wound repair, tissue regeneration and exerting anti-inflammatory effects. Abnormal TGF-β1 signaling can cause pathological fibrosis in response to tissue injury [56,57]. Moreover, the dysregulation between pro-inflammatory (IL-6) and anti-inflammatory (IL-10) cytokines can lead to hypertrophic scarring. In a study by Hedayatyanfard et al. [54], a 5% topical ointment losartan was investigated as a treatment for hypertrophic and keloid scars. The results showed that the application of losartan ointment led to significant improvements, including vascularity, pigmentation, pliability and height; at the end of treatment period, scars were smaller. Furthermore, patients reported a reduction in itching in the scar tissue following the application of losartan ointment [54].

Liao et al. [52] have found that Ang II is a major regulator for epidermal stem cells (ESCs). The administration of captopril, an ACEI or valsartan, an AT1R antagonist, resulted in the suppression of adhesion, migration and proliferation of human ESCs. On the other hand, blocking AT2R has opposite effects. These outcomes highlight the presence of a negative AT1R–AT2R cross-talk. As ESCs control skin turnover, interruption of Ang II signaling can lead to the disturbance of tissue self-renewal and wound healing [52].

## 7. The Implication of RAS in AIDD

### 7.1. Psoriasis

Psoriasis is a chronic immune-mediated disorder that impacts around 2% of the world’s population. It is characterized by the formation of erythematous, indurated, scaly and itchy skin plaques, which can often be painful. Moreover, it is associated with an elevated risk of developing various comorbidities, including psoriatic arthritis, diabetes mellitus, obesity, cardiovascular disease (CVD) and inflammatory bowel disease [58,59].

The risk factors associated with CVD, such as dyslipidemia, stress and smoking, frequently overlap as risk factors for psoriasis too. Systemic inflammation holds a significant role in the intricate connection between psoriasis and the development of atherosclerosis plaques. There is an observed pro-inflammatory phenotype in the endothelium. This includes the increased expression of IL-1β, VCAM1 and CXCL10. While numerous pro-atherogenic cytokines are elevated in psoriasis, it is worth noting that IL-6, IL-17, IFN-γ and TNF-α assume a prominent role in causing endothelial dysfunction and the development of atherosclerosis. Furthermore, there is an impaired production of vascular NO, which contributes to the occurrence of coronary microvascular dysfunction [60,61,62].

#### 7.1.1. Pathogenesis

Psoriasis is a skin condition associated with the IL-23/IL-17 pathway, an important factor in the disease’s development. Recent evidence suggests that Ang II can induce potent inflammation related to IL-17 [63].

The development and persistence of psoriatic inflammation can be attributed to disturbances in IIS within the skin. During the initial stages of psoriasis, myeloid dendritic cells secrete IL-12 and IL-23 and facilitate T-cell differentiation into distinct subtypes, Th1 and Th17 cells. Th1 cells produce IFN-γ and TNF-α, promoting the activation and proliferation of keratinocytes and facilitating the adhesion molecules expression. Th17 cells generate IL-17 and IL-22, promoting the angiogenesis within the lesion and the proliferation of keratinocytes [64,65].

Psoriasis involves an AI component, where the immune system mistakenly targets self-antigens. Autoreactive T cells specifically recognize and react against various self-antigens present in the skin. Some of the self-antigens implicated in psoriasis include keratins, the antimicrobial peptide LL37 and ADAMTSL5 [39].

**Figure 5:**
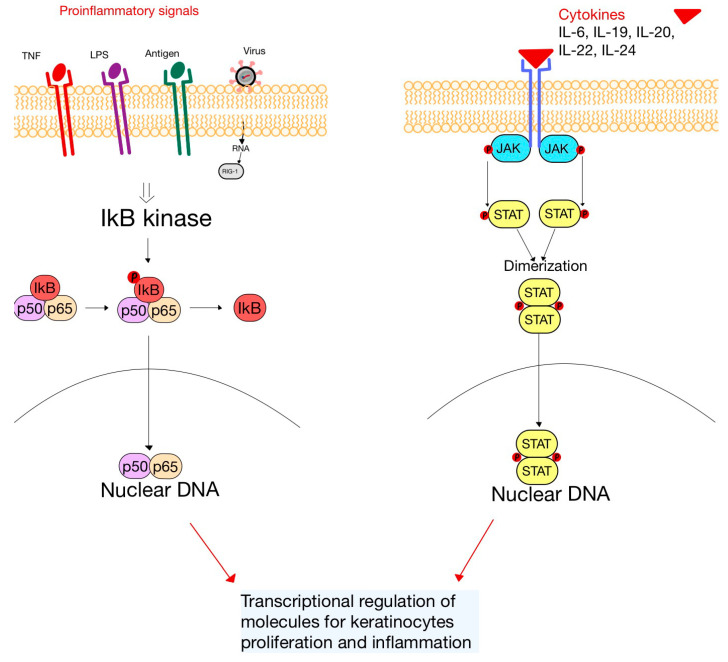
The altered signaling pathways in psoriasis. On the left side, the NF-κB pathway and on the right side, the JAK-STAT pathway. Both pathways lead to the proliferation of keratinocytes and inflammation (adapted after [66]).

The intricate mechanisms underlying the development of psoriasis can be attributed to disruptions in signal transduction pathways that play a role in modulating the activation and migration of immune cells (Figure 5). Furthermore, they also exert control over the viability, growth and differentiation of keratinocytes involved in psoriasis. Notably, the expression of NF-κB and STAT3 is found to be upregulated in psoriatic skin in comparison to healthy individuals [66].

The NF-κB signaling pathway can be triggered by numerous stimuli, such as TNF, IL-1, IL-17, LPS and viruses. Increased retinoic acid inducible-gene 1 (RIG-1), a major sensor for RNA viruses, has been observed in psoriatic skin lesions. It was discovered that the stimulation of the NF-κB pathway through RIG-1 is linked to the DCs’ generation of IL-23 during psoriasis. Cytokines (e.g., IL-6, -19, -20, -22 and -24) have been associated with the psoriasis pathogenesis and can induce the activation of STAT3 [66,67].

#### 7.1.2. ACE/AT1R Gene Polymorphisms

The ACE gene, situated on chromosome 17q23, comprises 25 introns and 26 exons [29]. There is a potential association between psoriasis incidence and polymorphisms in the ACE gene. One particular polymorphism within intron 16 involves an insertion (I) or deletion (D), with the D allele being linked to higher levels of ACE [51].

The severity of psoriasis and its related complications, including heart disease, hypertension and OS, appear to be influenced by the expression level of the AT1R gene. The AT1R-A1166C variant, specifically the C allele, is strongly correlated with OS and inflammation, making it a significant independent predictor [68]. According to Mohammadi et al. [69], in comparison with the levels in the control group, the prevalence of the C allele of AT1R was considerably higher among psoriasis patients [69]. Furthermore, in a study by Tanhapour et al. [29], there was found a noteworthy increase in the risk of psoriasis when individuals simultaneously possessed the C allele of AT1R A1166C and the I allele of ACE [29].

#### 7.1.3. ACEIs and ARBs

Antihypertensive drugs are commonly prescribed to patients not only for hypertension but also for conditions like arrhythmias, congestive heart failure, migraine, stroke and hemangioma. However, it is worth noting that the use of these medications may be linked to diverse cutaneous adverse effects, including the development of psoriasiform eruptions. A study conducted by Song et al. [70] suggests important associations between the incidence of psoriasis and the use of various medications, including antihypertensive drugs, such as ACEIs [70].

Substances modulated by ACEIs in the nerve system may contribute to the pathogenesis. In particular, SP, which is increased by ACEIs, appears to have stimulatory effects on keratinocyte proliferation, while upregulating IL-1, IL-8 and TNFα. In addition, vasoactive intestinal peptide (VIP), which is reported to be increased by lisinopril, stimulates proliferation and migration of human keratinocytes [71].

One hypothesis explaining the association between ACEIs use and psoriasis revolves around the role of bradykinin. ACE possesses dual catalytic domains and can interact with two distinct natural substrates: it cleaves Ang I and inactivates bradykinin. ACEIs have a greater affinity for the bradykinin binding sites than the Ang I, suggesting that these drugs predominantly inhibit the degradation of bradykinin. The high bradykinin levels caused by the use of ACEIs can induce vascular relaxation by triggering the release of prostacyclin, NO and epoxyeicosatrienoic acids. On the other hand, inhibiting the degradation of bradykinin by ACEIs leads to alterations in the kinin–kallikrein system, resulting in higher concentrations of inflammatory metabolites [19]. By inhibiting the degradation of bradykinin, ACEIs have the potential to activate the arachidonic acid cascade and elevate kinin levels and inflammatory cytokines. Notably, interleukins are linked to psoriasis and are recognized as key contributors to abnormal keratinocyte proliferation [70].

In a study by Zeini et al. [63], the potential positive effects of a topically applied 1% ointment of losartan were investigated in mice with imiquimod (IMQ)-induced psoriasis. As Ang II induces an inflammation related to IL-17, losartan mitigated the responses related to Th17 and reduced the levels of AT1R and Ang II. Moreover, the treatment with Losartan improves the clinical appearance and reduces the pathologic characteristics of skin inflammation induced by IMQ in mice [63].

#### 7.1.4. The Risk of Psoriasis in COVID-19

The skin has been implicated in the transmission of SARS-CoV-2 and it is believed that the integrity of the skin barrier could potentially influence the transmission of the virus through the skin [72]. SARS-CoV-2 invades the host cell by its spike interacting with ACE2R, present on the surface of cells [73]. ACE2 is present in the skin, and in psoriasis, there is an elevated expression of the enzyme [72]. In patients with psoriasis, IL-17–mediated inflammation is linked to elevated expression of ACE2 in skin plaques [74]. Treatment with IL-17 Abs demonstrated a decrease in expression of ACE2 in the skin, leading to the recovery of the skin barrier and alleviation of the inflammatory condition [72].

**Figure 6:**
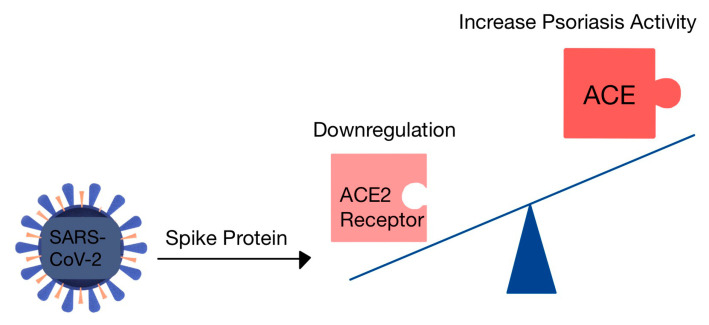
The role of ACE2R in psoriasis and COVID-19. The interaction coronavirus spike protein-ACE2R triggers the downregulation of ACE2. Thus, there will be an overproduction of Ang by the ACE (adapted after [75]).

The excessive activity of ACE (Figure 6) in patients with COVID-19 can exacerbate psoriasis and lead, in the known cases, to a higher occurrence of cardiovascular events. It is worth noting that elevated ACE activity is associated with more severe psoriasis and a higher prevalence of cardiovascular comorbidities among this population [75].

### 7.2. Systemic Sclerosis (SSc)

SSc is a rare AI disease that triggers fibrosis in the skin and subcutaneous tissue, affecting other organs, including the heart, kidneys, lungs and gastrointestinal tract. Among the most severe complications of SSc is scleroderma renal crisis, which is characterized by increased activity of plasma renin and acute kidney injury [76].

The heart is a pivotal organ affected by the disease, posing a heightened risk of sudden cardiac death. Heart complications encompass diastolic dysfunction, valvular issues, pericardial effusion, conduction block and the potential presence of myocardial fibrosis and inflammation. IL-1 plays a primary role in initiating the myocardial inflammatory process and in causing cardiac dysfunction [77,78,79].

The cause of SSc is unknown, involving genetic, environmental and immunological factors, as well as mechanisms related to cell senescence and inflamm-aging [80]. Up to 95% of patients diagnosed with SSc exhibit the presence of anti-nuclear Abs. Also, many individuals with the condition display specific Abs, including anti-centromere Abs (ACA), Abs targeting topoisomerase 1 (Topo-1 or Scl-70), and RNA polymerase III (RP3) Abs [81].

The disproportion between Ang II and Ang-(1-7) has been implicated in the pathogenesis of SSc, ACE2 being a key regulator of this balance. When the activity of ACE2 is compromised, ECs may lose their protective mechanisms, leading to dysfunction similar to what is observed in SSc. Anti-ACE2 Abs have emerged as another type of functional autoAbs that have the ability to disrupt the equilibrium between Ang II and Ang-(1-7). The anti-ACE2 Abs are not specific to SSc and likely arise due to the presence of polyautoimmunity, a condition that affects some individuals with SSc. While the prevalence of anti-ACE2 Abs is relatively low among SSc patients, their presence can have a significant impact on the depletion of plasma Ang-(1-7). This depletion, in turn, can promote an unfavorable phenotype within the microcirculation, potentially leading to adverse effects [82].

It is widely acknowledged nowadays that approximately 85% of SSc patients have autoAbs targeting the AT1R in their plasma. These Abs have the potential to trigger inflammation in the lungs and skin, dermal fibrosis and ECs apoptosis. Research has shown elevated levels of Ang II and endothelin 1 (ET1) in the tissues and blood of SSc patients. Kill et al. [83] demonstrated that IgG samples from patients diagnosed with SSc that were positive for anti-AT1R and anti-ET1 type A receptor (ETAR) Abs generate fibrotic and proinflammatory responses in ECs and fibroblasts from healthy donors by activating Ang and ET1-receptors. Both AT1R and ETAR play roles in regulating vascular function, ECM production, VSMCs proliferation and inflammatory responses.

**Figure 7:**
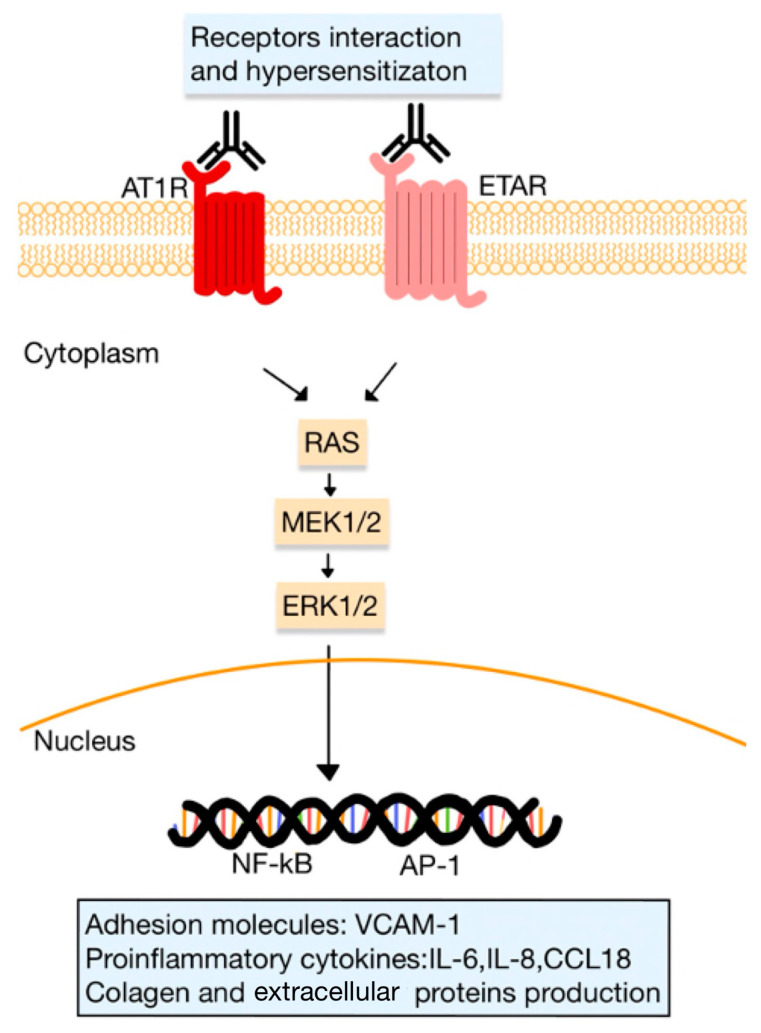
Functional autoAbs against AT1R and ETAR in SSc. The activated receptors initiate signaling pathways (involving kinases, such as RAS, MEK and ERK pathways), that regulate the transcription of various genes for adhesion molecules, proinflammatory cytokines and ECM proteins. The interaction between receptors, such as the heterodimerization of AT1R and ETAR, can lead to receptor hypersensitization (adapted after [9]).

It is presumed that Abs targeting AT1R and ETAR may participate in the development of SSc (Figure 7). Furthermore, the agonistic stimulation of AT1R on cells implicated in immunity, including monocytes, PMNs, B and T cells, induces the expression of pro-inflammatory genes (e.g., TNF-α, IFN-γ, IL-1, -6, -8, and -17). Chemokines, particularly MCP-1, play a significant role in attracting immune cells to the affected tissues, such as the skin. Activation of these receptors controls the trafficking of immune cells, including monocytes and PMNs, through chemotactic mechanisms, cytokine production and alteration of adhesion molecule expression.

Abs targeting AT1R and ETAR stimulate the expression of VCAM1 and induce the release of CCL18 or IL-8. These events lead to an elevated accumulation in the skin of inflammatory cells, including PMNs. Moreover, it is noteworthy that PMNs themselves express AT1R, and it was suggested that these specific Abs may activate AT1R on PMNs within blood vessels. The activation of PMN AT1R may direct these cells to areas of inflammation, where they can exacerbate tissue damage and contribute to the progression of the disease [9,51,80,83].

In animal models, it has been demonstrated that the presence of anti-AT1R Abs can induce SSc-like symptoms. This was observed in mice, in which either the production of anti-AT1R Abs or the injection of monoclonal anti-AT1R Abs resulted in the development of skin inflammation. In vitro experiments have revealed that anti-AT1R Abs can elicit specific responses in ECs. They induce the phosphorylation of ERK 1/2 and stimulate the expression of messenger RNA for TGF-β, VCAM-1 and IL-8 [80].

Studies investigating ACE polymorphisms have suggested that specific genetic variations are linked to SSc. According to Rodríguez-Reyna et al. [27], the prevalence of polymorphisms in the RAS genes and their potential with organ implication were examined in Mexican patients with SSc. In these patients, logistic regression analysis uncovered noteworthy associations between the AT1R-680 (rs275652) and AT1R-119 (rs275653) genetic variants, indicating a significant association with critical vascular dysfunction. These genetic variations are situated in the regulatory region of the AT1R gene, specifically on chromosome 3q21-25. However, it is important to note that this study presents certain limitations, such as a poor sample proportion and the absence of functional assays in order to evaluate the specific effects of the AT1R gene variation [27]. These limitations suggest the need for further research with bigger sample sizes and functional studies to elucidate the effects of AT1R gene variations on vascular manifestations in SSc.

### 7.3. Lupus Erythematosus (LE)

LE is an inflammatory AI disease that can affect multiple systems in the body (systemic lupus erythematosus, SLE) or isolated skin (cutaneous lupus erythematosus, CLE). The pathogenesis of SLE is complex and multifactorial, involving various contributors, such as OS, inflammation, immune stimulation, autoAb generation, overproduction of type 1 interferon (IFN) and tissue damage. Approximately 70% of individuals with SLE experience cutaneous lesions [84,85]. These lesions in SLE encompass a wide spectrum, ranging from a temporary acute malar rash, known as acute cutaneous lupus erythematosus (ACLE), to more severe and debilitating manifestations like discoid lupus erythematosus (DLE) or lupus profundus. These chronic lesions have the potential to cause disfigurement and significantly affect self-image and quality of life [86].

CVDs rank among the most significant contributors to disability and mortality in patients with SLE. There are various cardiac manifestations, such as myocarditis, pericarditis, atherosclerosis, valvular disease, arrhythmias and thrombosis. The equilibrium of vascular damage and protection mechanisms is disrupted due to an interplay involving OS, pro-inflammatory cytokines, B cells activation, the presence of autoAbs and abnormal T cell responses. Moreover, IFN 1 impairs the quantity and function of endothelial progenitor cells and exacerbates atherosclerotic lesions [87,88,89].

Research has identified a connection between variations in the ACE gene and the occurrence of SLE. Some studies suggest that these polymorphisms enhance ACE concentrations, which, in turn, could contribute to a higher incidence of SLE [90]. Individuals carrying the “D” allele face a greater risk of developing SLE [91].

The AT1R gene [92] and AT2R gene [93] polymorphisms might be considered as potential risk factors for children from Egypt with SLE [92,93]. Shoaib et al. [92] hypothesized that the AT1R gene polymorphism observed in SLE patients reduces the regulatory effect of miRNA155 on AT1R gene expression, leading to high levels of AT1R. This, in turn, contributes to enhanced pro-inflammatory effects. In addition, elevated serum ACE levels in SLE patients stimulate the production of Ang II, which acts through the AT1Rs and intensifies the pro-inflammatory effects, increasing the susceptibility to SLE [92].

Further studies made by Shoaib et al. [93] in Egyptian children marked the first exploration of the relationship between AT2R gene polymorphisms and SLE susceptibility. In SLE, the processes of clearance and/or apoptosis could be unstable. The presence of AT2R gene polymorphism could potentially impact AT2R activity, leading to disturbances in the clearance of cells and apoptosis, which may contribute to the susceptibility to SLE. Another plausible mechanism is that an atypical AT2R gene might attend to a low AT2R activity, thereby promoting the dominance of AT1R-mediated effects. This dominance, in turn, leads to an upregulation of pro-inflammatory pathways and the manifestation of fibrotic effects, both of which are distinctive characteristics of SLE. Furthermore, the study revealed significant elevations in serum levels of ACE among patients with SLE compared to the control group. Increased ACE levels in serum have been linked to augmented generation of ROS, which can induce OS and tissue damage in individuals with SLE [93].

Soto et al. [90] targeted the protective arm of the RAS as a prospective therapeutic approach in mice models with SLE. The therapeutics investigated included losartan, lisinopril and Mas agonists (Ang (1-7), NorLeu). The findings of their research demonstrated that Mas agonists were capable of reducing pathologies and alleviating immune changes in mice to a similar or even superior level as ACEIs/ARBs. Daily systemic administration of these RAS therapies demonstrated a significant reduction in the onset and severity of rash formation and paw swelling. Furthermore, histological analysis revealed a corresponding reduction in skin sections in hyperkeratosis and acanthosis. Immunological parameters were also observed, such as a decrease in circulating anti-dsDNA Abs, activation of T cells and reduction in lymph node size. The observed enhancement in both the quantity and quality of mesenchymal stem cells (MSCs), combined with a decrease in OS and inflammation, seems to have a contributory role in the reduction of SLE [90].

ACEIs slow SLE progression. This treatment approach leads to a significant reduction in the production of proinflammatory cytokines. These changes can be influenced by several factors, including dosage, the host’s characteristics and the specific nature of the disease. While captopril and lisinopril have been studied in both mouse models and human AI disease models, the exact mechanisms by which they exert their effects are not yet fully understood [94]. Another noteworthy study, conducted by Nocito et al. [95], presented the impact of captopril on IFN-I responses. They found that both systemic or per os administration of captopril reduced the responses of IFN-I. In addition, captopril treatment also demonstrated a decrease in classic clinical markers, including autoAbs levels and immune-complex deposition [95].

### 7.4. Vitiligo

Vitiligo is a frequent depigmenting skin condition with an approximated prevalence worldwide of 0.5–2%. This disorder is characterized by the loss of melanocytes, resulting in representative non-scaly and chalky-white macules. Various mechanisms have been proposed, such as genetics, autoimmunity, OS, inflammatory mediators and melanocyte detachment. In addition, vitiligo patients have a higher susceptibility to developing other AIDD, including LE and alopecia areata [96,97].

Patients with systemic vitiligo frequently exhibit metabolic disruptions. These individuals may face an elevated likelihood of developing atherosclerosis and dyslipidemia, potentially elevating their susceptibility to CVD [98,99].

A review, supported by a meta-analysis by Almohideb et al. [100], shows that there are associations of vitiligo with ACE gene polymorphism. Individuals carrying the I/D genotype exhibited a heightened susceptibility to vitiligo in comparison to those with the D/D and I/I genotypes. Additionally, the D/D genotype displayed a higher risk of vitiligo compared to those with the I/I genotype. In comparison with the D/I and D/D genotypes, the I/I genotype was demonstrated to have a protective influence against the susceptibility of vitiligo. Moreover, certain populations, such as Egyptians and Indians, have been reported to have a higher vulnerability compared to Europeans [100].

ACE contributes to the development of vitiligo through various mechanisms. This enzyme is responsible for deactivating bradykinin, regulating skin-neurogenic inflammation, and breaking down substance P and other neuropeptides. When the body experiences harmful stimuli like mechanical or chemical injuries, sensory nerves can release neuropeptides such as substance P, which in turn can trigger or amplify inflammatory reactions. These reactions include plasma extravasation, activation of leukocytes, cytokine production and mast cell activation. Also, the deactivation of bradykinin and the kallikrein–kinin system by ACE holds considerable significance in the inflammatory process [100,101].

Increased levels of IL-6 are reported in patients diagnosed with vitiligo. IL-6 promotes interactions between leukocytes and melanocytes by inducing the expression of ICAM-1 in melanocytes. This, in turn, leads to the activation of B-cells and the release of autoAbs, ultimately resulting in the destruction of melanocytes. The elevated production of IL-6 in vitiligo has been associated with melanocytic cytotoxicity. Moreover, previous studies have shown that NO can have a self-destructive effect on normal human melanocytes, impairing their attachment to the ECM and leading to depigmentation. NO, a highly reactive radical, can induce cellular toxicity by impairing metabolic enzymes. In vitiligo cases, the presence of 6 tetrahydrobiopterin, an important co-factor involved in NO synthesis, may contribute to increased NOS activity and subsequently higher levels of NO [30,102].

### 7.5. Alopecia Areata (AA)

AA is a common chronic AI disease characterized by hair loss, affecting approximately 2% of the population. AA patients may experience hair loss in specific areas, the entire scalp (including eyebrows and eyelashes) or even across the entire body. The disease pathogenesis is primarily driven by IFN-γ-mediated immune responses and the activation of cytotoxic CD8 T cells, which target hair follicles and nails [103,104,105].

It has been suggested that AA is associated with CVD. The proteomic blood profile in patients with AA reveals elevated levels of proatherogenic and inflammatory proteins [106,107]. Wang et al. [108] proposed that the occurrence of AA might be linked to cardiac remodeling, as indicated by elevated cardiac troponin I amount. However, their study is limited by the low population size, and more research is needed [108].

In a study by Fahim et al. [47], the ACE activity levels in the serum and skin of AA patients were examined to investigate the involvement of the local RAS in the development of this disease [47]. Researchers discovered associations between ACE and AA, suggesting a potential role of ACE in the pathogenesis of AA. Specifically, they observed higher serum ACE levels in more severe cases of AA, while the tissue levels of ACE were notably lower in AA patients compared to controls. It was proposed that Ang I might contribute to the inflammation seen in AA, leading to ACE consumption and reduced tissue levels of this enzyme. Further investigations are required to fully understand the potential involvement of the RAS in AA [47,109].

### 7.6. Pemphigus

AI pemphigus diseases encompass various entities with severe prognoses, known as pemphigus vulgaris (PV) and foliaceus (PF) [110]. PV, an AI blistering disease, has the potential to be life-threatening. Patients experience constant erosions and blisters caused by the detachment of keratinocytes (acantholysis), with the splitting of suprabasal epidermal layers. The underlying cause of pemphigus lies in the presence of immunoglobulin Abs targeting specific proteins on the cell surface of keratinocytes. Antigens (Ags) implicated in PV are known as desmogleins (Dsg) 1 and 3 [111,112]. Wada et al. [113] discovered that Ags, such as renin, unrelated to skin tissues, are coexpressed in mTECs (medullary thymic epithelial cells) that express Dsg3 [113].

PV has been associated with CVD. Numerous inflammatory pathways, such as IL-17, IL-22 and TNF-α have been demonstrated to be elevated in PV patients. Prolonged systemic activation of these pathways could potentially contribute to an increased risk of CVD. Also, cases of irregular cardiac rhythm and AI myocarditis have been reported [114,115,116].

Drugs can provoke or aggravate pemphigus. Thiol drugs like captopril were studied extensively in this regard. However, non-thiol drugs, including another ACEIs, such as enalapril, ramipril, fosinopril and ARBs, can also induce pemphigus. Treatment with ACEIs may trigger the formation of circulating autoAbs without apparent symptoms of the disease.

Research has demonstrated that ACEIs can induce, in approximately 52% of sera of individuals without pemphigus, Abs directed to Ags found in the superficial epidermal cells. The autoAbs generated by drug-induced PV or PF, such as with captopril, exhibit the same molecular-level antigenic specificity as autoAbs found in patients with PV/PF. In one reported case, ARBs, specifically candesartan and telmisartan, which are commonly prescribed as alternatives to ACEIs, were implicated as potential triggers or sustainers of PF. It is hypothesized that these drugs may induce keratinocyte adhesion loss and autoAbs production through indirect immune responses [110,117,118].

### 7.7. Bullous Pemphigoid (BP)

BP is a prevalent AI bullous disease distinguished by the presence of tense blisters on urticarial plaques, predominantly found on the trunk and extremities, and accompanied by severe pruritus. In BP are present autoAbs that recognize self-Ags in the basement membrane, specifically BPAG2 and BPAG1. These Ags contribute to the epidermis–dermis adhesion through the formation of hemidesmosomes [119].

Although BP has traditionally been considered primarily a skin disease, research has provided evidence of hypercoagulability and systemic inflammation. In addition, the presence of BP Ags in the myocardium may establish a significant link between BP and cardiac disease [120]. A study by Shen et al. [121] indicates that BP is linked to a five-fold greater risk of CVD mortality [121]. Moreover, Kalińska-Bienias et al. [122] demonstrated the correlation between arterial hypertension and BP. These findings suggest the need for further investigation [122].

ACEIs, mainly captopril, enalapril and ramipril, have been reported to cause BP. While PF is listed as a possible adverse reaction to lisinopril within the medication’s leaflet, BP is not. To our knowledge, there are two cases reported on lisinopril-associated BP [123,124].

There are two proposed mechanisms suggesting how ACEIs may induce BP. Firstly, they can activate and/or potentiate the pro-inflammatory kinin system by inhibiting the inactivating ACE. Secondly, ACEIs possess hapten-like properties, allowing them to bind to and modify proteins in the lamina lucida, which may trigger the production of autoAbs against these ‘neo-Ags’ [124].

Intriguing, Nozawa et al. [125] conducted the first study to reveal that lisinopril can prevent the development of dipeptidyl peptidase 4 inhibitor (DPP4I)-associated BP. DPP4Is are oral antidiabetic drugs used in the treatment of type two diabetes mellitus, but they increase the risk of BP, through unclear mechanisms. The Ang-(1-7)/MasR axis has emerged as a potential therapeutic target in DPP4I-associated BP. DPP4Is enhance Ang-(1-7) production by promoting ACE2 expression, while ACE and MasR expression remain unchanged. Furthermore, Ang-(1-7) stimulates through MasR the polarization of M2 macrophages; the production of MMP9 by M2 macrophages is implicated in the pathogenesis of BP. Lisinopril, identified through analysis of clinical data as a potential ACEI for the treatment of BP, was found to reduce the expression of MMP9 mRNA and the polarization of M2 macrophages induced by DPP4Is. These results provide valuable insights into the underlying mechanisms of BP and emphasize a promising target for the treatment [125].

### 7.8. Lichen Planus (LP)

LP is a chronic AI disease that typically affects the mucosae, skin and its appendages, with a potential risk for malignancy. The skin lesions associated with LP are usually self-limiting and sometimes itchy, whereas the oral lesions are long-standing and do not respond well to treatment [126].

Numerous CV risk factors are associated with LP: obesity, hypertension, dyslipidemia, impaired fasting glucose, metabolic syndrome and inflammatory markers. Elevated homocysteine levels were observed in individuals with LP, and this could potentially serve as an indicator for predicting CVD. Moreover, the polymorphism of the methylenetetrahydrofolate reductase gene might act as a potential risk factor for LP development, making these patients more susceptible to an increased risk of CVD [127,128,129].

The serum ACE activity was found to show a significant increase in patients with LP in comparison with healthy individuals [130]. In addition, ACEIs can induce lichen planus. There have been reported two cases suggesting that Captopril and Ramipril induced LP pemphigoides. Unfortunately, mechanisms are still unknown [131,132,133].

### 7.9. Other AI Diseases with Dermatological Implication

Dermatomyositis (DM) is an entity characterized by skin manifestations, including heliotrope and Gottron’s papules. Patients with DM have an increased CV risk for cerebrovascular accidents, ischemic heart disease and venous thromboembolism. Studies have shown that there is a correlation between the ACE D/D genotype and susceptibility to DM. Individuals carrying the D/D genotype exhibit elevated levels of ACE, while those with the I/I have the lowest levels. One possible hypothesis suggests that the ACE polymorphism favors the production of Ang II, a powerful proinflammatory modulator that enhances and sustains immune responses, as observed in many AI diseases [134,135].

The SARS-CoV-2 syndrome shares similarities with a rare AI syndrome called anti-melanoma-differentiation-associated 5 positive (aMDA5P) DM. Mecoli et al. [136] were the first to describe the prevalence and clinical characteristics of anti-ACE2 autoAbs in aMDA5P DM. The presence of these autoAbs could serve as a biomarker for serious disease and offer insights into the pathogenesis [136].

The ACE gene polymorphism also contributes to an increased predisposition to Behcet disease (BD). BD exhibits vascular manifestations such as arterial occlusions, the formation of aveurysms and pseudoaneurysms, occlusion of veins and Budd–Chiari syndrome. The cardiac involvement can manifest as myocarditis, pericarditis, valvular insufficiency, coronary arteritis, intracardiac thrombosis and sinus of Vasalva aneurysms. Possessing the D allele may pose a risk for BD [137,138,139]. According to Mandal et al. [140], the dominant model genotype (DD vs. ID and II) is associated with a 1.6-fold increased risk of BD [140].

## 8. Conclusions

This review has the purpose to rate the importance of RAS in AIDD. RAS has a vital role in mediating inflammation and the innate immune system, as presented above. Moreover, RAS components are expressed in the skin and can act separately from the plasma RAS. This tissue system influences its proliferation and differentiation, with implications for wound healing, scar formation and tissue remodeling.

By elucidating the particular mechanisms underlying RAS dysregulation, more studies are needed to develop new procedures that regulate the RAS to reduce inflammation, attenuate tissue damage and enhance the quality of life and clinical outcomes for patients with these challenging pathologies. RAS emerges as a promising avenue for exploration in AIDD. Elucidating its complex interactions within the skin microenvironment could pave the way for new therapeutic strategies. Nonetheless, further research is necessary to fully comprehend the complexities of the RAS and its contribution to AIDD.

## Data Availability

Not applicable.
